# Pathways to care: IDPs seeking health support and justice for sexual and gender-based violence through social connections in Garowe and Kismayo, Somalia and South Kivu, DRC

**DOI:** 10.1016/j.jmh.2022.100129

**Published:** 2022-09-05

**Authors:** Clayton Boeyink, Mohamed A Ali-Salad, Esther Wanyema Baruti, Ahmed S. Bile, Jean-Benoît Falisse, Leonard Muzee Kazamwali, Said A. Mohamoud, Henry Ngongo Muganza, Denise Mapendo Mukwege, Amina Jama Mahmud

**Affiliations:** a Centre of African Studies, Chrystal Macmillan Building University of Edinburgh, 15a George Square, Edinburgh EH8 9LD, UK; b Somali Institute for Development and Research Analysis (SIDRA), Garowe, Puntland, Somalia; c Faculté des Sciences Économiques et de Gestion, Centre d'Excellence Denis Mukwege (CEDM-UEA), Bukavu, South Kivu, Democratic Republic of the Congo; d Faculté des Sciences Agronomiques et Environnement; Centre d'Excellence Denis Mukwege (CEDM-UEA), Bukavu, South Kivu, Democratic Republic of the Congo; e Fondation Panzi; Centre d'Excellence Denis Mukwege (CEDM-UEA), Democratic Republic of the Congo

**Keywords:** IDPs, Displacement, SGBV, Somalia, DRC, Customary law, NGOs, Social connections, Pathways to care, Health systems, Justice, Gender, Panzi

## Abstract

A growing literature documents the significant barriers to accessing care that Internally Displaced Persons (IDPs) face. This study focuses on gender-based violence (SGBV), an issue often exacerbated in times of forced displacement, and adds to extant debates by considering the wide range of social connections (pathways and actors) involved in providing care beyond the formal biomedical (and justice) system. This research asks, who do IDPs turn to following SGBV and why? How effective do IDPs perceive these social connections to be? To answer these research questions, the study used ‘participatory social mapping’ methodology for 31 workshops held with over 200 participants in Somalia and the Democratic Republic of the Congo in 2021/2022. Pathways to SGBV-related care for IDPs appear eclectic and contingent upon not only the availability and accessibility of support resources but also social, cultural and gendered beliefs and practices. ‘Physical’, mental health, and justice needs are intertwined. They are hard to decouple as many actors cut across need categories, including family, faith and aid organisations, and customary institutions. Comparing Congolese and Somali sites of displaced communities, we see significant similarities and overlaps in pathways to care. While both countries have experienced severe erosions of state capacity, NGOs and parallel faith-based and customary legal, psychological, and health systems have filled the state's weakness to varying degrees of acceptance by IDP participants. A comprehensive understanding of the local milieu, which requires illuminating the logics behind where people actually turn to for care, is crucial for interventions supporting SGBV victims/survivors; indeed, they risk being inefficient if they only address barriers to formal systems.

## 1 Introduction

It has been established that, despite having the same citizenship status as their 'hosts', Internally Displaced Persons (IDPs) face significant barriers to accessing healthcare (Amodu et al., 2020), and have been facing diminishing health-related official development assistance (ODA) (Roberts et al., 2022). It is particularly the case for women (Amodu et al., 2020), some of whom are victims^1^ of sexual and gender-based violence (SGBV). The reasons for the lack of access to care for IDPs include, among others, poverty, adverse effects from displacement, and other disadvantages relative to host populations (Cantor et al., 2021). However, when IDPs are asked about their options in seeking care and where they *actually* turn to, they give a wide array of responses, which traverse the boundaries of formal and informal, indigenous and biomedical, state and non-state sources of healthcare and support. Taking a holistic view of care, our research utilizes the 'social connections' methodology to explore the perspectives and decision-making processes of displaced people and their neighbours when experiencing SGBV (Strang and O'Brien, 2017, Strang and Quinn, 2021). This methodology derives from our research questions: who do IDPs turn to following SGBV and why? How effective do IDPs perceive these social connections to be? We argue that answering these questions is essential to holistically address SGBV and thereby contribute to a growing literature of those investigating the complexity and systemic issue of SGBV in conflict and post-conflict areas. (Baaz and Stern, 2010, Anholt, 2016).

We focus on displaced people in the contexts of Somalia and the Democratic Republic of the Congo (DRC), two settings where access to healthcare is difficult for many and even more for IDPs (Mohamed et al., 2021), (Amisi et al., 2019). While considering the significant lack of resources and capacity of the formal healthcare and legal systems, we principally argue that to understand and address SGBV in IDP settings, biomedical and psychosocial healthcare and justice cannot be decoupled or compartmentalized. Victims and their families long for and seek justice simultaneously within and outside of the law to address health and mental health concerns of SGBV. By taking diverse pathways to care seriously, through NGOs, religious and indigenous healers, and customary authorities, we contribute to a broadening scope of wellbeing, biomedical and psychosocial healthcare. This article illuminates that IDP pathways to care are contingent upon cultural and gendered beliefs and practices, which help and hinder access to care and justice.

## 2 Background and rationale

The story of access to healthcare and justice relates to the state—or in settings of conflict and post-conflict, its incapacity. However, within this story, there is a myriad of non-state actors, institutions and support resources —which we call 'social connections'—that aid or impede access to healthcare and justice by replacing or syncretizing with the state. DRC and Somalia are textbook cases of what some academics and policymakers call 'failed', 'fragile', 'collapsed', or 'weak' states (Rotberg, 2004). Without wading deeply into this debate, we agree with others who claim that governance is more complicated than these terms and that 'stateness' is unevenly projected and negotiated amongst a myriad of actors even in conflict settings (Hagmann and Hoehne, 2009). With the weakening of the state due to conflict, Lake (2018) argues that despite the lack of state power in eastern DRC, the heavy presence of domestic and international human rights NGOs has paradoxically shaped judicial proceedings relating to SGBV in more progressive and effective directions comparatively to supposedly 'strong states' such as South Africa, where there is also high instances of SGBV. Yet in policy and academic literature on legal and justice systems and SGBV in conflict, most debates have not, until recently, engages directly with the question of healthcare provision (Lake, 2018, Manjoo and McRaith, 2011, Oo and Davies, 2021) made little research engagement in these settings until recently. The same seems to apply to healthcare research among IDPs and (post) conflict; there is limited discussion on linking health and justice (Cantor et al., 2021, WHO 2020). Our research contributes to a burgeoning field of research that seeks to transcend disciplinary approaches of SGBV by looking at the close connection between justice and healthcare (Liebling et al., 2020, Mastura et al., 2021). For us, this is the only way to reflect our data: when speaking to IDPs in communities experiencing widespread SGBV, these individuals and group discussions bring up health and justice in tandem because the absence of either for them means holistic healing and prevention of violence remain incomplete. In other words, we stress the importance of linking health and justice because it is important to our interlocutors and health and justice is mutually constitutive in their lives.

### 2.1 Context

Eastern DRC and Somalia have been subjected to protracted conflicts since the early 1990s, which have caused the displacement of millions, both within and across borders. These conflicts take place against the background of longer histories of violence marked by authoritarian rule and colonization. Numbers are difficult to verify but a 2017 World Bank report (World Bank Group, 2017) puts DRC and Somalia in the top six for most IDPs and states that most IDPs live in towns and cities (while 1% live in camps and 11% in self-settled camps). These settings affect IDPs' access to livelihoods and services. Those living close to urban areas have a greater chance of finding employment, whereas IDP encampments and settlements are more visible to humanitarians and receive higher proportions of funding and services relative to the populations in these settings (Fielden, 2008). However, as shown below, the displacement sites in DRC and Somalia blur the line between camps and integrated housing, urban and rural, and even displaced versus non-displaced. Moreover, whereas UNHCR takes the lead in supporting refugees, the IDP governance regime is more complicated. For instance, the World Health Organisation (WHO) is the health cluster lead for each country, and their website claims there are more than two hundred national, international and UN organizations in DRC and Somalia working on health.^2^ This proliferation of non-state actors points to a significant replacement of the state relating to healthcare services.

### 2.2 DRC

Much of Eastern DRC has been embroiled in regional and localized conflicts and violence since the fall of the Mobutu regime in the 1990s. The conflict has been continuously fuelled through a complex web of local armed groups mobilized around historic grievances relating to ethnicity, land rights, regional politics, and other issues. According to Internal Displacement Monitoring Centre (IDMC), by the end of 2020, there were more than 5.2 million IDPs.^3^ In South Kivu—the site of this research—recent internal displacements have been triggered by violence related to land, mineral resources, politics, and inter-communal tensions (Verweijen, 2020) and widespread flooding (UNHCR, 2020). Some estimates from 2015 say in South Kivu 96% lived with host families, while four percent lived in temporary settlements (Nguya, 2019); although these populations are highly transitory, the data is fragmented and contested, and the context has changed significantly since then. Eastern DRC, located far from the capital and political centre of Kinshasa, is characterized by fragile institutions; basic social services such as health facilities are often in very precarious situations, and some are supported by UN agencies and international NGOs (Lake, 2018, Seay, 2009). Moreover, Eastern DRC has been given the dubious moniker of the 'rape capital of the world'; however, without diminishing the prevalence of these crimes, we join Chloé Lewis and others who challenge this problematic discourse (Lewis, 2021).

### 2.3 Somalia

Somalia has seen multiple causes of displacement for more than thirty years through civil war, clan conflicts, failed foreign interventions from the United States and Ethiopia, the rise of al-Shabaab insurgency, as well as natural disasters such as floods, tsunamis, droughts, famine, and locust infestations. Today, there are nearly 3 million IDPs by the start of 2021, most of whom reside in southern Somalia.^4^ Most IDPs are displaced from rural areas to informal urban campsites where many lack the skills necessary to integrate and face significant poverty compared to host communities (OCHA, 2021). These challenges are particularly acute for marginalized groups who face even greater structural exclusion. As a result of the aforementioned conflicts and disasters, the Federal Government has limited funding and capacity to provide services, and nearly half of the population of about 7.7 million people needs humanitarian assistance (OCHA, 2021). The Somali Ministry of Health's capacity to oversee and regulate healthcare services and providers is extremely limited (Ahmed et al., 2020), and SGBV is a prevalent problem in IDP contexts in Somalia. Still, it is underreported due to the stigma associated with sexual violence (Save the Children, 2019).

The cities of Kismayo and Garowe have been chosen as field sites in Somalia. Kismayo is a strategic port city in Jubaland State in southern Somalia. Kismayo, like South Kivu, has active combatants where government and regional forces are fighting with al-Shabaab, which controls significant land areas near the city. The city is controlled by Jubaland military forces, a heavily armed police force, and Kenyan soldiers of the African Union Mission to Somalia (AMISOM). Moreover, in addition to conflict, Kismayo has faced significant climate change-induced desertification, leading to acutely strained livelihood opportunities and high eviction rates. These factors all intersect with a significantly eroded state capacity for ensuring health and justice for IDPs and hosts (UNEP-IOM, 2021). Garowe is the capital of Puntland State and, due to its relative peace and security, has attracted IDPs from the southern part of Somalia and returnees and refugees from Ethiopia (The World Bank Group, 2014).

## 3 Methods and materials

The social connections methodology was designed to map and understand the people and organisations displaced people turn to in times of need. It seeks to find who members of a community turn to as a resource when they need help or support, and how. The methodology, which was pioneered by Strang and O'Brien (2017) and Strang and Quinn (2021) who supported the methods training and initial rollout of this research, can be used for a wide range of issues. ‘Help’ and ‘support’ can take different shapes, from meeting material needs to addressing grievances. For the present project, we started by asking workshop participants to identify and discuss (on flipchart) who and where people in their community go to if they experience deep sadness, persistent physical pain, or SGBV. We then discussed the mapping with them. The present article focuses on SGBV, although discussing social connections for deep sadness and physical pain intersected with SGBV in conversations. Our primary material is the notes of the discussions during the workshops and the list of social connections they generated. The works of Strang and O'Brien (2017) and Strang and Quinn (2021) provides more details on the theoretical foundations –among others, social capital theory– and methodological details of the social connections methodology. Recognising that not all social connections are of equal importance and influence, this article relies on qualitative discussions among workshop participants probing into the levels of importance of each social connections (another possibility, which we do not cover in the present paper, is to have a large representative survey based on the workshops). The research team was composed of international and national researchers working for University of Edinburgh, L'Université Evangélique en Afrique (UEA), and the Somali Institute for Development Research and Analysis (SIDRA). In the DRC, some are also affiliated with Panzi Foundation, a key provider of support for victims of SGBV. While the research was not carried you on behalf of Panzi, this affiliation may have affected researchers’ interaction with research participants. We reflect upon this positionality later in the article.

### 3.1 Research sites

Our research covers rural and urban sites, including four sites in the DRC and five sites in Somalia. They are described in Table 1 below. The sites were chosen to ensure a diversity of settings based on key socio-spatial features that are known to reflect displaced site characteristics and affect local integration: rural/urban or peri-urban; spatially segregated apart from host population either as a camp or new neighbourhood; different housing structures (formal/informal, tents/solid structures); and type, length and reasons for displacement (recent/prolonged violence/natural disaster).

**Table 1:** field site characteristics.

IDP site name	Location of site	Cause of displacement	Site characteristics
*DRC*			
Katana	Located in Kabare territory 40km north of city of Bukavu	Displaced by violence	Integrated amongst long-term residents in large rural town;
Kavumu	Located in Kabare territory 40km north of city of Bukavu	Displaced by violence	Integrated amongst long-term residents in large rural town;
Katogota	Located south of Bukava along highway on the way to city of Uvira	Displaced by violence	Land was set aside for camp in 2009 for IDPs; maintained by humanitarian actors
Uvira	Site located on the outskirts of Uvira, the second largest city in South Kivu	Displaced by recent flooding	Urban IDPs with houses constructed by international humanitarian actors
*Somalia*			
Fanole	Located inside the city of Kismayo, Jubaland	Civil war in 1991; conflict with al-Shabaab; droughts/floods	Informal structures made of tents, plastic sheets, and other temporary shelters.
Midnimo	Located on outskirts of Kismayo, Jubaland	Created in 2017 for refugees returning from Kenya; Others displaced by conflict, droughts, floods	Formal IDP settlement with permanent houses made of stone/brick and iron roofs. Has its own health centre, school, women's centre, police station, shops
Shabelle	Located on the outskirts of Garowe, Puntland	Created in 2000 for those displaced by conflict, droughts, floods in Shabelle, Somali Region of Ethiopia.	Informal structures made of tents, plastic sheets, and other temporary shelters.
Bilan	Located inside the city of Garowe, Puntland	Created in 2005 for those displaced by tsunami in 2004	Formal settlements no longer considered camp; it has completely integrated into the city
Jilab	Located on the outskirts of Garowe, Puntland	Created in 2010, initially built as a camp for those living informally in area and for those displaced by drought	No longer considered a camp but has integrated into a village and integrated into the city

### 3.2 Workshop methodology

During the workshop discussions (conducted in Swahili and Somali), participants were asked: ‘If someone in your community has been struggling to cope with everyday life more than most people for a long time due to SGBV (to the point where it significantly interferes with their ability to sleep, work/make money, take care of their children, attend social events/groups, etc.), who would they speak to about it and who and where could they ask for help?’. Each of the terms was chosen as general phrases and carefully translated into corresponding Swahili and Somali ‘idioms of distress’ (Carroll and Murug, 2004, Greene et al., 2016, Im et al., 2017), rather than direct translations due to inconsistent levels of awareness and understandings of biomedical and psychosocial diagnoses in these communities. Asking the questions hypothetically, rather than if the participants have directly experienced these situations was a deliberate ethical decision to attempt to avoid bringing up traumatic or distressing experiences in individuals. While these three questions guided the workshops, the researchers were trained to ask probing questions about each social connection, exploring these support resources and people's perceptions and motivations for turning to them. They also initiated prompts to elicit more social connections by asking the guiding questions in different ways.

Seven women and five men in the DRC and six women and six men in Somalia were trained online in the workshop methodology alongside role-playing practice and piloting. The Somalia team also conducted pilot workshops among IDPs in Mogadishu. Each workshop included at least three researchers: a facilitator, flipchart recorder, and field note taker. The workshops were divided by gender, where the researchers’ genders matched the participants to enable more open discussions on such sensitive topics. People were more open to talk when the workshops were divided between over and under 30-year-olds, although not all workshops were organised this way.

### 3.3 Research participants

Prior to facilitating the workshops, the research team contacted local authorities to gain approvals and recruited research participants over 18 years old through local leaders or ‘gatekeepers’ who hold authority in the field sites. For the DRC, this entailed gaining clearance from the heads of localities (*groupements*) or villages and, in some instances, the territorial administrator, and participants were recruited through local facilitators. The size of the samples and the selection of participants through our local gatekeepers mean that the workshops are unlikely to be properly representative of the population. However, the researchers worked with the participant recruiters to select widely across age, gender, wealth, and situation of displacement. We collected basic socio-demographic data at the beginning of each workshop to ensure that we indeed had participants coming from a wide range of backgrounds. The primary aim of the workshop was to reach a saturation point of listing social connections until no new connections were discussed. By combining different workshops with different key demographics in each site, we aimed to generate a broad picture of the main SGBV-related connections in each site. The displaced and non-displaced binary is often subjectively identified and contested (Nguya, 2019): in the field sites in the DRC, there were both displaced and non-displaced participants because displaced populations were integrated into communities of non-displaced populations^5^ whereas in Somalia, only IDPs were included because they were part of spatially segregated camps. In total, there were 111 participants in the DRC, with 94 displaced (49 men vs 45 women) and 17 non-displaced (7 men vs 10 women). In Somalia, there were 178 total IDPs. Across all the sites, there were 95 men and 83 women. Appendix A provides further detail below.

### 3.4 Ethics and safeguarding

Recognising that research about health conditions among displaced populations is highly sensitive, the project has taken multiple ethical and safeguarding precautions detailed in Appendix B.

### 3.5 Data analysis

**Fig. 1:**
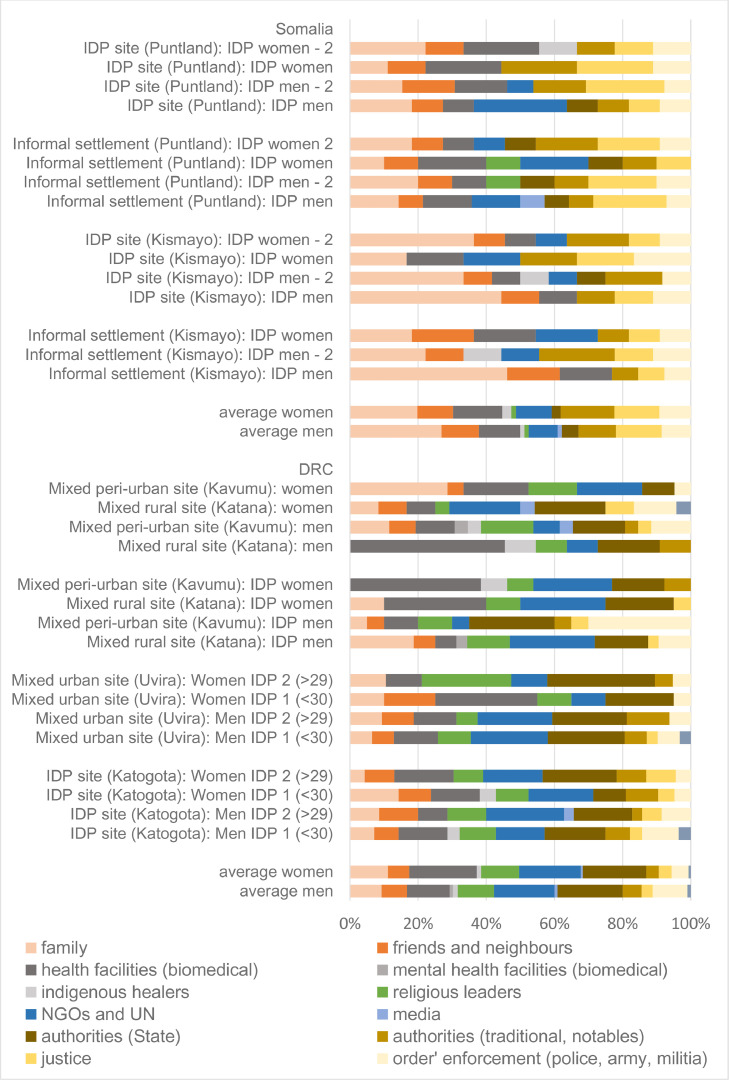
Social connections categories by field site.

The researchers present in the workshops recorded field notes, and this data was deductively coded and analysed (using the Nvivo 20 software). Every workshop was coded to find participant quotes and field notes pertaining to SGBV. All social connections relating to SGBV were coded with health, mental health, and justice nodes as well as formal and informal connections. Iterative discussions throughout the analysis and writing period were important because members of the writing team, the lead author included, were not from these locations and the Somali and Congolese authors clarified and corrected potential biases or lack of understanding of local context. Once coded, the writing team analysed and discussed the coded material and the pathways to care, which is presented below. The quantitative data was recorded from the flipcharts used in the workshops into a spreadsheet, which created frequency data for how many workshops mentioned each social connection. These social connections were then grouped into the categories featured in Fig. 1.

## 4 Results/Discussion

### 4.1 Quantitative data analysis

Fig. 1 below sums up the connections mentioned in each workshop. Connections are grouped into categories for the sake of clarity. It is worth noting that participants in workshops in the DRC usually mentioned a lot more connections than Somalia (23.5 on average versus 10.5). There are a few possible hypotheses from this. This includes the high numbers of aid organisations that directly provide aid in South Kivu and the higher levels of literacy there (see participants’ profiles in Appendix B). It is also useful to note that workshops in sites that are ‘not mixed’, i.e. only count IDPs, led to identifying fewer connections, but only marginally so (when we had workshops with both displaced and non-displaced people in the DRC, a similar number of connections were identified). The main difference in terms of the number of connections that hold across the two countries is based on gender: men cite more connections than women (26.8 vs 20.1 in the DRC; 11.4 vs 9.5 in Somalia). A possible hypothesis is that men are less confined to the home sphere (both countries struggle with gender parity at many levels). Perhaps more interesting is to consider the nature of the connections rather than their mere number, which is what we do with Fig. 1. It is not clear that gender is a decisive factor, but a few key trends are visible:

Firstly, across the board, specialised psychological support is almost inexistent –as already pointed out in some of literature (Ibrahim et al., 2022). Specialised psychiatric and psychosocial services are few in the two contexts, but they exist. However, they do not appear a ‘natural’ connection when participants are probed about gender-based violence. It is, however, important to note that in the DRC, Panzi hospital effectively includes psychological support as part of its ‘holistic’ approach (Mukwege and Berg, 2016); it appeared in almost every workshop (but is counted as ‘biomedical health facility’). As we explain later, psychological support may be provided by religious figures in the DRC, while family may play a substantial role in Somalia.

Secondly, biomedical infrastructures (including, but not at all limited to, Panzi Hospital) take a central place in the DRC, and this aligns with some of the recent efforts of NGOs to ‘mainstream’ gender-based violence support in health facilities (see background section). In Somalia, they are much less systematically present (again, in the case of SGBV, they are cited when the focus of the workshop is on health in general).

Thirdly, justice tends to be emphasised more heavily in Somalia, where both the formal justice system –including the police– and local authorities and notables are key connections. In contrast, participants in the DRC were more likely to turn to other types of authorities, from chiefs to local administrators and Members of Provincial and National Parliaments. They are technically justice arbitrators but do, in practice, play such roles (Muhire, 2017).

There are clear limitations with our quantified data. They do not tell us anything about the reliability of the connection or the frequency they are ‘connected with’. They also do not distinguish between the connections that people find useful and trustworthy and those they feel compelled to use because of social pressure. A good example is the prominence of the family and clan justice system in Somalia, which many of the women of our workshop felt frustrated about. For these reasons, and to better understand the dynamics at play, we now turn to our qualitative material. We discuss the findings alongside the presentation of the results rather than in a separate section.

### 4.2 Understanding social connections: qualitative data analysis

#### 4.2.1 Framing SGBV and pathways to care

There is a range and severity to types of SGBV, which trigger differing pathways to care and must be addressed separately. In our study, the most frequent forms of SGBV—which are by no means always separate or discrete categories—include domestic SGBV where the perpetrator is a member of the household or family, SGBV perpetrated by community members outside of the immediate family network, and finally, SGBV perpetrated by armed combatants, which usually inflicts the most severe physical violence. This last form of SGBV is a more dominant feature in the DRC than in Somalia. The bulk of the workshop dialogue focused on sexual violence toward women with rarer instances of sexual violence toward men—which triggers wholesale different community responses and reveals clear gendered differences toward care and justice in these contexts. Unlike the DRC, the Somalia sites also frequently mentioned female genital cutting as a form of SGBV, which will only be minimally discussed because it has a more complicated level of acceptance than the other forms of SGBV described above.

We conceptualise each pathway to care—proximal, healthcare, mental health, and justice—in a wide fashion by incorporating informal, indigenous, religious, or customary actors rather than narrowly assessing formal, biomedical and psychosocial treatment and legal forms of care and justice (Kirmayer and Bhugra, 2009). We take an inclusive approach because this reflects the lived reality of the populations in this study. In constrained environments, people turn to many social connections (see Fig. 1) and simultaneously pursue multiple pathways to care, as we demonstrate below. In our discussions, the participants listed going to many forms of support and care, including herbalists or *waganga,* which is Swahili for or ‘witch doctors’; Christian and Muslim religious leaders and healers; or even savings and loan groups for physical ailments, mental health treatment, and justice.

#### 4.2.2 Proximal social connections

Not surprisingly, the first people whom victims turn to—or those who incidentally witness acts of violence—are people within the closest proximity to the victim:

“the people who send the victims to the hospital or the health centre are either relatives, the closest neighbour, a friend or just a person of good will, her sisters or brothers”.^6^

This quote from a woman in Kavumu (DRC) reflects a general finding: proximal connections are often the initiators of care pathways. Many people reported that neighbours heard acts of violence and intervened by transporting the victims to receive healthcare or reported incidents to the police. One participant from Garowe recalls such a situation:

“A woman was attacked and raped at midnight. The neighbours heard her scream and came out to help. Recognising the man who committed the rape, they contacted the camp committees and together reported the case to the human right defender's office. A lawyer was hired for the case, and the victim was treated at the hospital, ending in arrest of the offender.”^7^

People set off on different pathways to care through proximal social connections in divergent ways: where one woman will immediately tell her parents or friends of a violent event, a different woman will try to hide these events out of shame or fear of stigmatisation. A group of women in Katogota discussing a hypothetical domestic rape situation acknowledged these dilemmas:

“Woman 1: ‘You go to a friend because talking with someone frees you up and makes you feel better.

Woman 2: ‘I think we should tell the mother who is the president of the church because at least she can't tell everyone in the village your secret because she is wise and God-fearing.’

Woman 3: ‘I think it's best to turn to your parents because they will always be there with you despite your decision.’

Woman 4:‘A neighbour—’

[People in the group yell and interrupts and says the neighbour is a bad idea because they will tell everybody your secret.]”^8^

Others acknowledged people they would turn to but have either passed away or were separated through displacement:

“My parents could help me, but they have already passed away. I look around, I don't have anyone to help me, so I trust in God. But if I were a member of a group, it could help me.”^9^

The groups she is referring to are saving groups known as *Mutuelles de Solidarité* (MUSOs), which offer informal meetings to access needed credit and finance outside of the formal banking system. Additionally, they support mental health wellbeing through friendships and solidarity.

Yet as the justice pathway section below analyses, proximal connections may also impede a victims’ access to justice in unsatisfactory ways for the victim, as argued in recent related literature on gender and refugee resettlement (Phillimore et al., 2021). Furthermore, proximal connections may be the perpetrators of violence. Unlike SGBV caused by community members or armed combatant, domestic violence occurs within proximal relationships, which causes a different pathway for a victim. Whether it is domestic violence from a husband or unequal economic relations within a household, many women spoke of husbands providing social, emotional, and economic deficits rather than capital as recounted by a woman in Katana:

“The behaviour of our husbands *zinatutesa* [they torment us]. I resort to my in-laws summoning my husband for advice, but he does not listen. I think of going away, but I find that I cannot abandon my children, so I just pray to God.”^10^

#### 4.2.3 Medical care

In all field sites, the participants acknowledged that following rape requiring an immediate attention, such as repair to bleeding, fistulas, or prolapses of organs, healthcare is readily available through local health centres, who refer to public hospitals if necessary. Despite the costs, victims and their families often find ways to treat for severely life-threatening or potentially debilitating injuries due to the severity of the injuries. People are unable or unwilling to seek treatment for not immediately life-threatening symptoms, such as STIs or bodily harm from assault. There is recognition in both countries that specialist care is needed. A man in Kismayo recalled the event of a young girl who was raped and taken to Kismayo General Hospital but rued the fact that there was “no unit in the hospital to provide specialist treatment and care for rape victims like her.”^11^ Likewise in the DRC, there is awareness of the need for quick treatment after sexual violence. A woman in Katogota recalled:

“the first advice I can give is to go to the hospital because we have always been told that if you are raped and you go more than three days without being treated, the disease can catch you.”^12^

The hospital referred in the quote above is Panzi Hospital, located in Bukavu, the largest city in South Kivu. Throughout our South Kivu workshops, both men and women frequently referred to Panzi Hospital, Panzi Foundation, or they directly mention the founder, Dr. Denis Mukwege, as the primary provider of health, mental health, and legal support. For example, when asked where people subjected to rape go, a woman in Katogota said simply, “I'm going directly to Mukwege.”^13^ Panzi Hospital was founded in Bukavu in 1999 and services were expanded through the Panzi Foundation in 2008. Dr. Mukwege, a gynaecologist, gained international fame through Panzi's work treating victims of sexual violence. This eventually led Dr. Mukwege to jointly receive the Nobel Peace Prize with Iraqi Yazidi human rights activist Nadia Murad in 2018. Panzi is a unique institution across our two contexts because it attempts to be a ‘one stop centre’ where they have developed four pillars of care consisting of medical, psychosocial, legal, and socio-economic reintegration interventions.

We focus on Panzi because it is exceptional compared to most conflict and post-conflict settings due to how well-funded it is and the wide range of services beyond formal medical care it provides. However, no singular organization can completely counter these challenges. Despite a more consistent state apparatus presence than the DRC, in Kismayo and Garowe, pathways to care are ad hoc and disjointed, unlike the Panzi Foundation's ‘one-stop’ model. That is not to say this work addressing these issues is not happening: Garowe has recently built a forensics lab to aid in rape convictions, and Human Rights Defenders were frequently cited by participants as a trusted source. In Kismayo the Jubaland Commission for Refugees and IDPs (JUCRI) leads on these issues.

In both countries, there are three principal reasons for not seeking treatment: stigmatisation, fear of retribution, and poverty (those reasons are widely discussed and in line with the main academic debates, see for instance Davies et al. (2016)). If it becomes known that somebody was a victim of rape, they will face stigma and may be divorced or unable to marry later. One discussion among a men's workshop in Kismayo described a seven-year-old who was raped and taken to the hospital. This event became known in the community, and afterward, “the victim faced stigma, she could not attend school with other children, so her family kept her at home. She became quiet and sad.”^14^ On the other hand, not seeking treatment can also lead to stigma. A woman in Kavumu explained that a victim could go discreetly for treatment or else their situation can be discovered: “one can feel bad when one is a victim of sexual violence, and in this case, I can go to a doctor for treatment instead of staying at home and hiding so as not to be humiliated in the neighbourhood.”^15^ Others are afraid to seek health treatment if their abuser is an intimate partner or family member due to threats of further violence. Finally, even though Panzi Foundation operates mobile clinics across different South Kivu health zones, and subsidizes other rural health centres, poverty and the cost of taking victims to hospital are prohibitive, even if there are no hospital fees. For example, in Kavumu one woman spoke of her difficulties finding the care she needs: “I would like to go to the Panzi Foundation or to Women for Women, but they are not easily accessible. I don't have the capacity to access them. I don't have the capacity to go there. Even to go to state institutions, you need money.”^16^ In the same community, a woman participant disclosed that she was a victim of domestic violence from her husband, yet she experiences such acute poverty that she claims she would willingly accept violence from her husband if she had enough money for food and other necessities.^17^

#### 4.2.4 Mental health pathways

Some of the literature argues that displaced populations lack an understanding of mental health or are reluctant to seek treatment out of fear of stigmatisation (Cavallera et al., 2016, Piwowarczyk et al., 2014, Byrow et al., 2020). However, in the social connections workshops, there was a near-ubiquitous understanding that victims of sexual violence require mental health support following violent events. One man in Kavumu stated it succinctly:

“mental assistance is needed to enable all these victims to return to their normal lives. The use of psycho-clinicians to help these people has been widely considered.”^18^

As described in the previous section, participants instead pointed to significant geographic and financial barriers to accessing this care. Despite the difficulty in access, people seek other pathways.

In South Kivu, Panzi Foundation was frequently cited as an attainable source of psychosocial support at no cost to the patient (apart from transportation). The foundation has set up residential treatment centres for sexual violence survivors who are stigmatized in their communities; facilitated recreational events to promote relaxation and healing; counselled victims’ family members; and trained other healthcare providers and police officers in trauma-sensitive care. Despite Panzi's outreach, many of these services are centralized in Bukavu. Outside the city there is a dearth of trained mental health practitioners and workshop participants stated more rural psychiatric centres are needed.^19^ In Somalia, although there is a general limitation of available trained mental health workers (Ahmed et al., 2020), the participants in Garowe and Kismayo were aware of and even mentioned by name specialist psychosocial and neurological health care clinics nearby and as far afield as Mogadishu. However, very few people can pay the fees and transport costs of these private clinics.

Despite lack of access to mental health services across rural South Kivu and Somalia, the participants pointed out the other forms of emotional support they receive. People turn to pastoral care from religious leaders or through the support of MUSO groups that they are a part of. Panzi Foundation recognised not just the economic benefits of MUSOs but also the mental health benefits of these groups. They train and equip women who leave their programmes to start these groups. We argue, however, that another key component of mental health for victims is accessing justice in ways determined by the victim, which is difficult in these settings with the absence of the state and deeply entrenched gender hierarchies.

#### 4.2.5 Justice pathways

*DRC:* The workshop participants were openly critical of the state's ability to attain justice. A woman in Uvira said: “there are always cases of rape in Uvira but unfortunately the state does nothing, armed robbers can even injure one, but the state will do nothing.”^20^ A woman in the same workshop recognizes it should be the state's role, but when asked where they would recommend a victim of SGBV to go they said:

“I *would* advise them to go to the state, but we know that the state will not give any help.”^21^

Consistent with Lake (2018) and others’ findings that there is a proliferation of NGOs in eastern DRC which often replace state judicial functions, the DRC workshops listed a significantly larger number of NGOs they turn to in comparison to the Somalia sites. For example, Panzi Foundation's legal pillar operates several legal clinics across South Kivu to accompany victims through the legal process. They also disseminate information through radio and community meetings about local, national, and international jurisprudence. There are other human rights organizations in the area who were mentioned as trusted source for legal support such as *Groupe Jérémie*, which is associated with the Catholic Church. However, as with medical care, even though these services are free, if people live remotely, then geographic and financial barriers obstruct uptake in services.

What makes persecuting perpetrators of rape in South Kivu difficult is when a member of an armed group carries it out. Often the perpetrator is not identifiable; and even if they are recognized, the authorities will likely not seek a confrontation with these violent groups. One notable exception includes a landmark military trial in 2017, which reached international attention, funding, and legal expertise. A provincial lawmaker Frederic Batumike and ten men in the militia he financed were convicted for the abduction and rape of young girls in the area (Perissi and Taquet, 2018). Most cases never make it to court, however.

Beyond the state and the proliferation of NGOs, in postcolonial settings, particularly in Africa, there are long histories of customary or parallel authorities and legal systems which share, contest, and negotiate legal authority (Mamdani, 1996). In the DRC, while customary courts step into the state void and maintain significant authority in land tenure and other cases, they are legally prohibited from weighing in on instances of SGBV (Baaz and Stern, 2010, Zongwe, 2012). Despite this prohibition there are oft-cited instances of rape, which are mediated outside of courts between families or through chiefs of their respected ethnic groups. When a woman is sexually assaulted, instead of taking it to court or punishing the perpetrator, a bride-price is negotiated. The victim is wedded to the perpetrator despite the DRC's national law specifying this as a crime that clearly violates the rights of the victim (Baaz and Stern, 2010, Zongwe, 2012). Similar processes are often followed when sexual assault is carried out in a domestic relationship. In a men's discussion of this scenario in Katogota, some participants thought the victim of assault should go to the police or legal clinics, whereas others said, “the uncle can also help resolve the situation.”^22^ Discussing the same topic in a women's discussion in Uvira, a site manager who was dominating the discussion forcefully spoke to this process of arbitration where the justice system is the last resort:

“if you are raped by your husbands, it is better first to address the in-laws and church members. And if the husband doesn't change, you have to go to the chief of the village, then to the police and even to the human rights office. It's a whole process.”^23^

Finally, in the absence of justice following cases of rape or murder, some respondents—predominantly men—described the process of revenge if judicial or customary justice is not reached. This can include the use of a hitman or armed group or also through witch doctors who use witchcraft with the intent to harm and kill alleged perpetrators.

*Somalia:* While Somalia is frequently included with the DRC in the ‘failed state’ narrative, the presence of the state and non-state actors differs significantly from South Kivu, which influences the pathways to justice victims seek in Garowe and Kismayo. Unlike in South Kivu, fewer non-state actors who offered legal services were mentioned (see Fig. 1). There is a palpably thick state presence in the two field sites in Somalia, albeit in differing ways. Garowe is the capital of Puntland State, a semi-autonomous Federal Member State, which has its own constitution, administers its own police and military forces, and has higher literacy rates and lower poverty levels than other regions Somalia (Stremlau, 2019). While IDPs in Garowe still face barriers to care, there is also access to health clinics and human rights defenders. Discussants in the Garowe workshops noted that state organizations were offering legal services through the human rights defender's office, which provides information and advice and representation to victims through lawyers or paralegals. However, there were varying degrees of awareness and ability to access from the IDP sites. What legal services were available in Kismayo were offered by JUCRI. Alternatively, Kismayo, the provisional capital of Jubaland State, is surrounded by al-Shabaab-controlled territory and is heavily securitized with a sizable presence of Jubaland police forces, federal military soldiers, and AMISOM soldiers. As such, people in Kismayo felt there was a better chance of police apprehending a perpetrator, whereas people in Garowe felt there was more support by the state available for courts and jurisprudence. Indeed, one man at Shabelle camp in Garowe lamented that there was no police state located nearby the camp to apprehend perpetrators of sexual violence.^24^

Despite the visible presence of the state, across Somalia there is a parallel, yet often entwined informal or customary law based on clan networks, which heavily influences justice outcomes following SGVB. Known as *Xeer* in Somali, this system is typically based around compensation from the offending parties through family or extended clan network to the family of the victim. This is commonplace even though it is prohibited by some laws such as the Puntland Rape act, which forbids tribal elders from resolving rape offences (Stremlau, 2019). Schlee (2013) argues that *xeer* should not be seen as preserving the role of equal justice, but rather is a form of bargaining for paying damages between clans or subclans, which often have significantly differential powers and resources to bargain. Moreover, women's ideas of justice are left out of the equation, as we demonstrate below. Whether or not the police and courts are brought into the process following SGBV is fluid and negotiable. The workshop participants in both cities provided myriad examples of how customary law replaced or worked with formal legal systems.

This system of compensation does not instil a sense of justice for many of the women in the discussion groups. They decried that money or in-kind goods are given to male relatives and never the victim.^25^ Another woman in Kismayo, reflecting on a rape case of a young girl resolved through clan elders said:

“when a case like this happens, the traditional leaders take over the case, and the case is not taken up by the rule of law agencies. This needs to change. The perpetrators must get harsh punishment so that it will be a lesson for those who are inclined to do similar horrible crimes.”^26^

This gendered sense of justice is laid bare when a man or boy is subject to rape or sexual violence. In these instances, the *xeer* system of adjudication is usurped, and police and courts are immediately involved.

This is not to say that women or people seeking change are passive to this system. There has been an emerging trend in Garowe of social media campaigns of people trying to apply pressure on local authorities and police to prosecute rape offences. In recent incident in Shabelle camp, people contacted a well-known television news reporter to document and share the case to provide further pressure, which may point to possible space for positive change. This is more difficult to do in Kismayo, however, where the state significantly censures the media.

## 5 Limitations

This research is exploratory in nature, while it provides a general sense of SGBV-related social connections in our field sites, it is not a comprehensive or even a representative study and information such as the reliability of frequency of relying on certain social connections need to be interpreted carefully. Relying on gatekeepers to recruit workshops participants was the only viable option but it has potential to bias discussions. It should also be noted that the study starts from a question (who do people turn to?) that will echo actual experience for those who have been victims of SGBV, but that same question is more hypothetical for other participants who have not been victims of SGBV (although some may have been close witnesses). This, and the workshop format, mean that our findings reflect a form of group consensus on what are seen as the SGBV-related social connections rather than the actual lived experience of survivors of SGBV (both are interesting in their own right but potentially different).

## 6 Concluding remarks

This article has covered significant ground by analysing people's (expected) trajectories after being subjected to various forms of SGBV. In line with a growing literature, our findings suggest that the way to begin to heal victims following SGBV comprehensively is to sync pathways of health, mental health, and justice (Liebling et al., 2020). As the literature on the social determinants of health insists, public health must be bolstered by law in order to support health systems and vice versa (Gostin et al., 2019). This is true also for cases of SGBV. However, our study also points out that while legal and justice systems are sometimes empowering for women (Gangoli, 2020), they are also sometimes discriminatory in contexts such as the DRC and Somalia. The need, as the literature suggests, is for proper survivor-centred justice to be put in place (Oo and Davies, 2021, Gangoli, 2020). This requires a comprehensive understanding of the local milieu, which requires illuminating the logics behind where people actually turn to for care and recognising the complexity of SGBV. It is not enough simply to address the barriers to formal systems.

Internally displaced populations, as our research has shown, should not be treated homogenously: the connections of those living in camps in Somalia and those living in peri-urban areas in South Kivu are very different –as we just emphasised, only an understanding of the local milieu can meaningfully inform responses. The limited data we have on the so-called host population suggest that the number and intensity of connections were, in our case, the main differences between IDPs and hosts (with IDPs worst off). In both Somalia and the DRC, displaced populations kept relying on customary and formal authorities as well as health services, and in contexts of generalised conflict (or at least low intensity violence) they access humanitarian assistance provided by institution such as Panzi in the DRC or JUCRI in Somalia. There remain a few areas that we will tackle in forthcoming research, they regard among others, the intensity of the IDPs social connections and will require establishing the situation among the non-displaced population first.

The positive ‘silver-lining’ to state ineffectiveness in South Kivu is that it has created an opening for Panzi and other local and international organisations to progressively influence health care and justice, which supports Lake (2018) thesis of positive gains toward SGBV human rights in the DRC. We take particular note of Panzi Foundation because it has developed an intimate knowledge of the local context, which it has accordingly used to implement its ‘one-stop’ logic of working with local authorities to incorporate medical, psychosocial, legal, and financial care to victims of SGBV, irrespective of whether they are displaced or not. We are not saying that Kismayo and Garowe and other contexts need their own Panzi Foundation, as that organization spawned out of its own history and context and captured international attention and aid that is unlikely to be easily replicable. Nor do we normatively vilify customary law and local authorities that remain key connections for IDPs and ‘host’ population alike, despite the women interlocutors’ deep dissatisfaction with these compensatory forms of justice. They contend that these systems maintain the status quo and foreclose the possibility of justice. Instead, we argue that any intervention made locally or from international actors must begin by understanding a holistic picture of the social connections. It is especially important to include these actors in a funding environment where IDP health has faced diminishing funding levels (Roberts et al., 2022). Aid could and should be localised for the sake of aid efficiency as well as efficacy to not neglect important social connections whom are often excluded from aid. Any process of reform must necessarily seek integration of pathways and connections across state and non-state institutions to make lasting positive change.

## Declaration of Competing Interest

The authors declare the following financial interests/personal relationships which may be considered as potential competing interests, Some co-authors are affiliated with the Panzi Foundation, which is a key provider of support to SGBV victims

## Figures and Tables

**Table:** 

		Somalia		DRC	
		Kismayo	Garowe	Katana	Uvira
Gender	Male	51%	56.1%	52%	49%
	Female	49%	43.9%	48%	51%
Age	years (average)	37.5	34	34	29
Marital Status	Divorced	6.3%	9.8%	4.2%	1.6%
	Married	69.8%	78%	65%	47.5%
	In partnership			6.3%	15%
	Single	16.7%	9.8%	21%	33%
	Widowed	7.3%	2.4%	8.3%	3.3%
Level of education	can't read and write	44.8%	40.2%	8.3%	8%
	can read and write	8.3%	25.6%		
	(some) primary	14.6%	22%	16.7%	19%
	Quranic School	20.8%	1.2%		
	(lower) secondary	1%	1.2%	37.5%	42.9%
	(some) high school / technical	8.3%	9.8%	29.2%	29%
	University	2.1%		8%	2%
Duration of Displacement	years (average)	7.8	10.5	18	7

## Footnotes

1 Though we recognise these concepts are contested and unsettled among academics and practitioners, the term ‘victim’ was most used by our participants; ‘survivor’ denotes somebody who has gone through wraparound treatment or somebody subjectively feels like they have overcome the violent event.

2 According to the WHO website. See https://healthcluster.who.int/.

3 See https://www.internal-displacement.org/countries/democratic-republic-of-the-congo for more details.

4 See https://www.internal-displacement.org/countries/somalia for more details.

5 For this research, we allowed participants to self-identify as displaced or non-displaced.

6 Woman FGD, Kavumu, 19/08/2021.

7 Man FGD, Shabelle, 24/01/2021.

8 Woman FGD, Katogota, 25/08/2021.

9 Woman FGD, Katana, 20/08/2021.

10 Woman FGD, Katana, 20/08/2021.

11 Man FGD, Kismayo, 10/01/2022.

12 Woman FGD, Katana, 25/08/2021.

13 Woman FGD, Katogota, 26/08/2021.

14 Man FGD, Kismayo, 10/01/2022.

15 Woman FGD, Kavumu, 19/08/2021.

16 Woman FGD, Kavumu, 19/08/2021.

17 Woman FGD, Kavumu, 19/08/2021.

18 Man FGD, Kavumu, 18/08/2021.

19 Man FGD, Katana, 20/08/2021.

20 Woman FGD, Uvira, 27/08/2021.

21 Woman FGD, Uvira, 27/08/2021.

22 Man FGD, Katogota, 26/08/2021.

23 Woman FGD, Uvira, 28/08/2021.

24 Man FGD, Shabelle, 24/01/2021.

25 Woman FGD, Kismayo, 12/01/2022.

26 Woman FGD, Kismayo, 12/01/2022.

## References

[bib0001] Cantor D., Swartz J., Roberts B., Abbara A., Ager A., Bhutta Z.A., Blanchet K., Madoro Bunte D., Chukwuorji J.C., Daoud N., Ekezie W., Jimenez-Damary C., Jobanputra K., Makhashvili N., Rayes D., Restrepo-Espinosa M.H., Rodriguez-Morales A.J., Salami B., Smith J. (2021). Understanding the health needs of internally displaced persons: a scoping review. J. Migr. Health.

[bib0002] Roberts B., Ekezie W., Jobanputra K., Smith J., Ellithy S., Cantor D., Singh N., Patel P. (2022). Analysis of health overseas development aid for internally displaced persons in low- and middle-income countries. J. Migr. Health.

[bib0003] Amodu O.C., Richter M.S., Salami B.O. (2020). A scoping review of the health of conflict-induced internally displaced women in Africa. IJERPH.

[bib0004] A. Strang, O. O'Brien, Mapping social connections, trust and problem-solving among conflict-affected populations, (2017) 72.

[bib0005] Strang A., Quinn N. (2021). Integration or isolation? Refugees’ social connections and wellbeing. J. Refug. Stud..

[bib0006] Baaz M.E., Stern M. (2010).

[bib0007] Anholt R.M. (2016). Understanding sexual violence in armed conflict: cutting ourselves with Occam’s razor. Int. J. Hum. Action.

[bib0008] Mohamed A.A., Bocher T., Magan M., Omar A., Mutai O., Mohamoud S.A., Omer M. (2021). Experiences from the field: a qualitative study exploring barriers to maternal and child health service utilization in IDP settings Somalia. IJWH.

[bib0009] C. Amisi, P. Adzande, S. Pothiawala, SGBV in camps in the DRC: Preliminary Results, 2019.

[bib0010] Rotberg R.I. (2004).

[bib0011] Hagmann T., Hoehne M.V. (2009). Failures of the state failure debate: evidence from the Somali territories. J. Int. Dev..

[bib0012] Lake M. (2018).

[bib0013] Manjoo R., McRaith C. (2011). Gender-based violence and justice in conflict and post-conflict areas. Cornell Int. Law J..

[bib0014] Oo P.P., Davies S.E. (2021). Access to whose justice? Survivor-centered justice for sexual and gender-based violence in Northern Shan state. Glob. Stud. Q..

[bib0015] WHO (2020).

[bib0016] Liebling H., Barrett H., Artz L. (2020). South Sudanese refugee survivors of sexual and gender-based violence and torture: health and justice service responses in Northern Uganda. Int. J. Environ. Res. Public Health.

[bib0017] Mastura A., Clemence D., Strelan P. (2021). Relationship between experiences of systemic injustice and wellbeing among refugees and asylum seekers: a systematic review. Aust. Psychol..

[bib0018] World Bank Group, Forcibly Displaced: Toward a Development Approach Supporting Refugees, the Internally Displaced, and Their Hosts, Washington, D.C, 2017.

[bib0019] A. Fielden, Ignored Displaced Persons: The Plights of IDPs in Urban Areas, UNHCR, Geneva, 2008.

[bib0020] J. Verweijen, Why Violence in the South Kivu Highlands Is Not ‘Ethnic’ (And Other Misconceptions About the Crisis), Kivu Security Tracker, 2020. https://blog.kivusecurity.org/why-violence-in-the-south-kivu-highlands-is-not-ethnic-and-other-misconceptions-about-the-crisis/.

[bib0021] UNHCR, Massive floods in DRC's South Kivu impact 80,000 people, kill dozens, (2020). https://www.unhcr.org/uk/news/briefing/2020/4/5e9ea96f4/massive-floods-drcs-south-kivu-impact-80000-people-kill-dozens.html (Accessed 9 December 2021).

[bib0022] Nguya G. (2019).

[bib0023] Seay L. (2009).

[bib0024] Lewis C. (2021). The making and re-making of the ‘rape capital of the world’: on colonial durabilities and the politics of sexual violence statistics in DRC. Crit. Afr. Stud..

[bib0025] OCHA (2021).

[bib0026] Ahmed Z., Ataullahjan A., Gaffey M.F., Osman M., Umutoni C., Bhutta Z.A., Dalmar A.A. (2020). Understanding the factors affecting the humanitarian health and nutrition response for women and children in Somalia since 2000: a case study. Confl. Health.

[bib0027] Save the Children (2019).

[bib0028] UNEP-IOM, Identifying climate adaptive solutions to displacement in Somalia, 2021, 2021.

[bib0029] The World Bank Group, Analysis of Displacement in Somalia, Washington, D.C, 2014. https://openknowledge.worldbank.org/bitstream/handle/10986/21056/932380WP0P12640t0DC0edits009012014.pdf?sequence=1&isAllowed=y.

[bib0030] Carroll J.K., Murug W. (2004). Gini: expressions of distress in refugees from Somalia. Prim. Care Companion J. Clin. Psychiatry.

[bib0031] Greene M.C., Ventevogel P., Tol W.A. (2016). Mental health and psychosocial wellbeing in congolese refugee survivors of gender- based violence: a desk review. UNHCR.

[bib0032] Im H., Ferguson A., Hunter M. (2017). Cultural translation of refugee trauma: cultural idioms of distress among Somali refugees in displacement. Transcult. Psychiatry.

[bib0033] Ibrahim M., Rizwan H., Afzal M., Malik M.R. (2022). Mental health crisis in Somalia: a review and a way forward. Int. J. Ment. Health Syst..

[bib0034] Mukwege D., Berg M. (2016). A holistic, person-centred care model for victims of sexual violence in Democratic Republic of Congo: the Panzi hospital one-stop centre model of care. PLoS Med..

[bib0035] Muhire B. (2017). Legal pluralism, customary authority and conflict in Masisi, (Eastern) Democratic Republic of Congo. J. Sociol. Dev..

[bib0036] Kirmayer L.J., Bhugra D., Salloum I.M., Mezzich J.E. (2009). Psychiatric Diagnosis: Patterns AndProspects.

[bib0037] Phillimore J., Pertek S., Alidu L., Mora C., Piper N. (2021). The Palgrave Handbook of Gender and Migration.

[bib0038] Davies S.E., True J., Tanyag M. (2016). How women’s silence secures the peace: analysing sexual and gender-based violence in a low-intensity conflict. Gend. Dev..

[bib0039] Cavallera V., Reggi M., Abdi S., Jinna Z., Kivelenge J., Warsame A.M., Yusuf A.M., Ventevogel P. (2016).

[bib0040] Piwowarczyk L., Bishop H., Yusuf A., Mudymba F., Raj A. (2014). Congolese and Somali beliefs about mental health services. J. Nerv. Ment. Dis..

[bib0041] Byrow Y., Pajak R., Specker P., Nickerson A. (2020). Perceptions of mental health and perceived barriers to mental health help-seeking amongst refugees: a systematic review. Clin. Psychol. Rev..

[bib0042] Perissi D., Taquet E. (2018). https://www.ijmonitor.org/2018/02/the-kavumu-trial-complementarity-in-action-in-the-democratic-republic-of-congo/.

[bib0043] Mamdani M. (1996).

[bib0044] Zongwe D.P. (2012). The new sexual violence legislation in the congo: dressing indelible scars on human dignity. Afr. Stud. Rev..

[bib0045] Stremlau N. (2019). Governance without government in the Somali territories. J. Int. Affairs.

[bib0046] Schlee G. (2013). Customary law and the joys of statelessness: idealised traditions versus Somali realities. J. East. Afr. Stud..

[bib0047] Gostin L.O., Monahan J.T., Kaldor J., DeBartolo M., Friedman E.A., Gottschalk K., Kim S.C., Alwan A., Binagwaho A., Burci G.L., Cabal L., DeLand K., Evans T.G., Goosby E., Hossain S., Koh H., Ooms G., Roses Periago M., Uprimny R., Yamin A.E. (2019). The legal determinants of health: harnessing the power of law for global health and sustainable development. Lancet.

[bib0048] Gangoli G. (2020). Gender-based violence, law, justice and health: some reflections. Public Health Ethics.

